# Mild traumatic brain injury increases vulnerability to post-traumatic stress disorder in rats and the possible role of hippocampal DNA methylation

**DOI:** 10.3389/fnbeh.2025.1539028

**Published:** 2025-03-03

**Authors:** Yujie Niu, Zhibiao Cai, Junkai Cheng, Jie Zhou, Xiaodong Qu, Changdong Li, Zhongjing Zhang, Shenghao Zhang, Yaqiang Nan, Qifeng Tang, Lei Zhang, Yelu Hao

**Affiliations:** ^1^Department of Neurosurgery, The 940 Hospital of PLA Joint Logistic Support Force, Lanzhou, Gansu, China; ^2^The First Clinical Medical College of Lanzhou University, Lanzhou, Gansu, China; ^3^Department of Neurosurgery, Xijing Hospital, Fourth Military Medical University, Xi'an, Shaanxi, China

**Keywords:** mild traumatic brain injury, post-traumatic stress disorder, vulnerability, behavior, hippocampus, DNA methylation

## Abstract

**Introduction:**

Clinical studies have established that patients with mild traumatic brain injury (mTBI) are at an increased risk for developing post-traumatic stress disorder (PTSD), suggesting that mTBI increases vulnerability to subsequent PTSD onset. However, preclinical animal studies investigating this link remain scarce, and the specific biological mechanism through which mTBI increases vulnerability to PTSD is largely unknown.

**Methods:**

In this study, we modeled mTBI in rats using a mild, closed-head, weight-drop injury, followed 72 h later by exposure to single prolonged stress (SPS) to simulate PTSD. Then, we investigated the impact of mTBI on subsequent PTSD development by observing the behaviors of rats in a series of validated behavioral tests and further explored the possible role of hippocampal DNA methylation.

**Results:**

We found that, compared with rats in the PTSD-only group, those in the mTBI + PTSD group exhibited higher anxiety levels, higher depression levels, and impaired spatial learning and memory as determined in the open field test, the forced swimming test, and the Morris water maze test, respectively. Rats in the mTBI + PTSD group also exhibited higher hippocampal DNMT3b protein expression compared with those in the PTSD group.

**Conclusion:**

In conclusion, our results demonstrated that mTBI increases vulnerability to PTSD in rats, possibly through alterations in hippocampal DNA methylation patterns.

## 1 Introduction

Comorbid mild traumatic brain injury (mTBI) and post-traumatic stress disorder (PTSD) have received increasing clinical attention over recent years due to the high rates of mTBI-PTSD co-occurrence among military personnel (Zhang et al., 2021). Clinical studies have shown that patients with mTBI are at an increased risk for developing PTSD, suggesting that mTBI increases vulnerability to subsequent PTSD onset (Loignon et al., 2020). Given the high comorbidity between mTBI and PTSD, it is crucial to understand the mechanisms through which mTBI enhances vulnerability to PTSD to develop effective preventative treatments. Preclinical studies can contribute to a better understanding of the interaction between mTBI and PTSD and can provide important insight into the underlying mechanisms (Schindler et al., 2021). However, preclinical animal studies investigating mTBI and PTSD remain scarce (Ojo et al., 2014), and the biological mechanisms through which mTBI promotes vulnerability to PTSD are largely unknown (Balasubramanian et al., 2021). In this study, we explored the impact of mTBI on subsequent PTSD development using the rat as a model. mTBI was induced by a mild, closed-head, weight-drop injury, followed 72 h later by PTSD simulation through exposure to single prolonged stress (SPS). Subsequently, the behaviors of rats were evaluated in a series of validated behavioral tests, and the potential role of hippocampal DNA methylation in mediating the increased vulnerability to PTSD following mTBI was explored.

DNA methylation, a form of epigenetic regulation, is mainly regulated by DNA methyltransferases (DNMTs) (Balasubramanian et al., 2021). DNMTs can be broadly characterized into two categories, namely, maintenance DNMTs, such as DNMT1, which methylate hemimethylated DNA; and *de novo* DNMTs, such as DNMT3a and 3b, which methylate previously unmethylated CpG sites (Zovkic and Sweatt, 2013). DNA methylation has been implicated in the development of both mTBI and PTSD (Nielsen et al., 2019; Balasubramanian et al., 2021). One study demonstrated that DNMT3b function in the hippocampus was upregulated 30 days after repeated mTBI (Balasubramanian et al., 2021), while another revealed that cued and contextual fear conditioning increased the expression of DNMT3a in the brain (Morris et al., 2014). In the current study, we investigated the possible role of hippocampal DNA methylation in mediating the mTBI-induced increase in vulnerability to PTSD by measuring DNMT1 and DNMT3b protein expression in the rat hippocampus.

## 2 Materials and methods

### 2.1 Animals

A total of 56 pathogen-free male Sprague–Dawley rats (approximately 6 weeks old and weighing 210 ± 20 g) were purchased from the Laboratory Animal Center of Lanzhou Veterinary Research Institute, Chinese Academy of Agricultural Sciences (Lanzhou, China) and utilized as subjects. All rats were housed under specific-pathogen-free (SPF) conditions in the SPF-level Animal Laboratory of the Medical Experimental Center, School of Basic Medical Sciences, Lanzhou University (Lanzhou, China). The rats were housed under controlled conditions (22°C, 50% relative humidity, 12-h:12-h light/dark cycle). Food and water were supplied *ad libitum*. The rats were housed 3–4 per cage, except during the social isolation stage of the SPS protocol. All efforts were made to minimize the number of animals used and the suffering of the animals.

### 2.2 Experimental design

**Figure 1:**
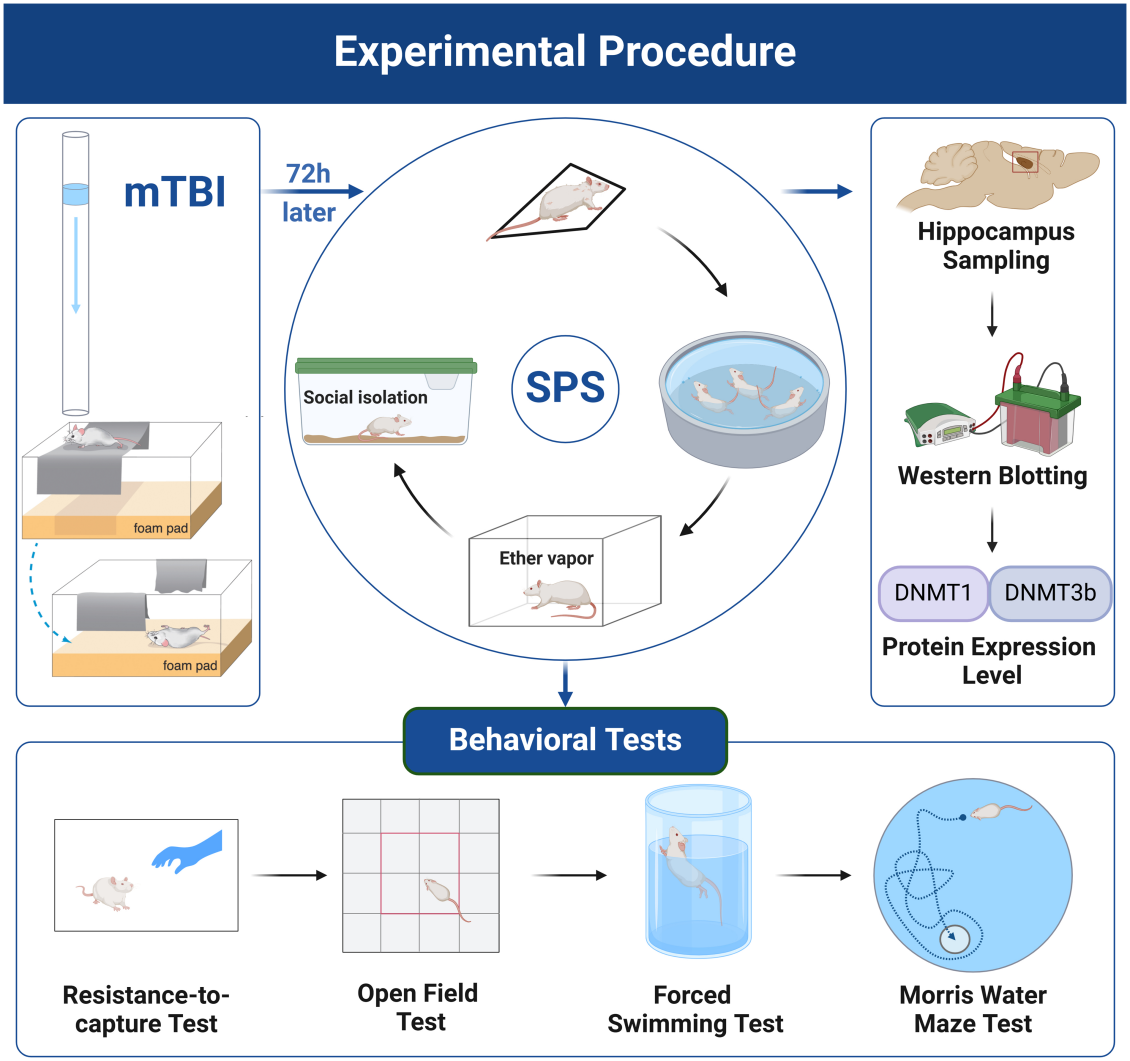
Experimental procedure. Created with BioRender.com. Images reproduced with permission from (Bodnar et al., 2019; Verbitsky et al., 2020).

On arrival, the rats were habituated to the animal facility conditions for 7 days before any experimental procedures. After habituation, the animals were randomly assigned to the following four experimental groups: control, PTSD, mTBI, and mTBI + PTSD (*n* = 14 per group). mTBI was generated using the mild, closed-head, weight-drop injury model, while PTSD was induced by exposure to single prolonged stress (SPS). In the mTBI + PTSD group, mTBI was induced first, followed 72 h later by PTSD. After modeling, six rats in each group were decapitated, and the bilateral hippocampus was rapidly dissected and stored at −80°C for western blotting analysis. The remaining eight rats in each group were subjected to a series of behavioral tests. The experimental procedure is shown in Figure 1.

### 2.3 Mild traumatic brain injury procedure

mTBI was induced using the non-surgical, closed-head, weight-drop technique as previously described (Hehar et al., 2015; Fraunberger et al., 2020). In brief, the rat was minimally anesthetized with isoflurane until the toe-pinch response was eliminated. The rat was then placed, chest down, on a scored piece of aluminum foil suspended approximately 10 cm above a foam pad. A 150-g weight was dropped from a height of 0.5 m through a plastic guide tube aiming to hit the head at approximately −2.5 mm relative to bregma. This produced a glancing blow and propelled the rat through the aluminum foil, ultimately landing in a supine position on the foam pad. Immediately after the injury, the rat received a topical administration of lidocaine at the site of impact and was then placed in the supine position in a clean warm cage for recovery. Rats not receiving the mTBI (control and PTSD groups) underwent a sham injury involving anesthetic exposure and placement on the weight-drop device, but did not receive the head blow.

### 2.4 Single prolonged stress protocol

Rats were exposed to SPS as previously described (Lisieski et al., 2018; Piggott et al., 2019). Briefly, SPS consisted of a sequential series of stressors—physical restraint, forced swim, ether anesthesia, and, finally, social isolation for 1 week. Rats were first individually restrained using cylindrical clear plastic restraints for 2 h in a prone position in clean cages with fresh bedding. Immediately following the restraint, groups of 3–4 rats were put together in a single Plexiglass cylinder (35 cm diameter × 60 cm height) filled to a depth of 40 cm with fresh water (25°C) for a 20-min forced group swim. After the swim, the rats were towel-dried and allowed to recuperate for 15 min in clean cages with fresh bedding. Subsequently, the rats were exposed to diethyl ether vapor until they lost consciousness. Finally, the animals were single-housed in a standard home cage for 7 days of social isolation. This 7-day “sensitization period” is necessary for the establishment of this model. Rats that were not exposed to SPS (control and mTBI groups) remained group-housed. All the rats were undisturbed during these 7 days.

### 2.5 Behavioral tests

Rats underwent a sequential series of behavioral tests in the order of resistance-to-capture test (RTC), open field test (OFT), forced swimming test (FST), and Morris water maze (MWM) test. Rats were acclimated to the testing room for at least 1 h before beginning the test procedures, except for the RTC test, which was conducted in the animal room. All behavioral tests were performed between 08:00 and 16:00 h.

#### 2.5.1 Resistance-to-capture test

The RTC test, which was used to assess the irritability of the rats, was performed as previously described (Yozgatian et al., 2008). The test was conducted in the animal room as the rats were being transferred to new cages to undergo the subsequent behavioral tests. The RTC was assessed in a single trial, during which the resistance of the animals to being picked up by the examiner (who was blinded to the experimental design) was scored. The level of resistance was scored as follows: 0, easy to pick up; 1, vocalized or shied away from the hand; 2, shied away from the hand and vocalized; 3, ran away from the hand; 4, ran away and vocalized; 5, bit or attempted to bite; and 6, launched a jump attack.

#### 2.5.2 Open field test

Anxiety-like behaviors and locomotion were studied by conducting the OFT. The apparatus comprised a black square base, 75 × 75 cm, with opaque 40-cm-high walls; a digital camera was suspended overhead. The central region of the open field arena was determined by dividing the base into 16 identical squares and selecting the four most central ones. Each rat was placed in the center of the apparatus and left to freely explore the arena for 10 min. After each trial, the floor of the apparatus was cleaned with 75% ethanol to eliminate the odor of the previous animal. Each test was recorded using a computer-assisted video tracking system (Techman, Chengdu, China). The total distance moved, the percentage of distance moved in the central area, and the percentage of time spent in the central area were measured.

#### 2.5.3 Forced swimming test

The FST was performed to evaluate depressive-like behavior in the rats (Nie et al., 2021). The test was conducted by individually placing each rat in a transparent Plexiglas cylinder (20 cm diameter × 50 cm height) filled with water (25°C) to a depth of 35 cm. Each rat was gently placed in the middle of the cylinder for 6 min and allowed to swim or float. Immobility was defined as the rat floating passively in the water without struggling or climbing and only making those movements necessary to keep its head above water. The rats were then towel-dried and returned to their home cage. The cylinder was rinsed and replaced with fresh water for each animal. Each test was recorded and the immobility time during the last 4 min was measured manually by a trained observer blinded to the treatment conditions.

#### 2.5.4 Morris water maze test

The spatial learning and memory of the rats were assessed by the MWM test, a classical test used for measuring cognitive performance (Andersen et al., 2019; Nie et al., 2021). In brief, testing was performed in a round water tank (1.2 m in diameter, 0.5 m in height) filled with water at 25°C. A 10-cm round transparent platform was hidden in a constant position in the tank submerged 1.5 cm below the water level. The quadrant where the platform was located was defined as quadrant Q. Within the testing room, only distal visuospatial cues were available to the rats for the localization of the submerged platform. Rats performed four trials per day to find the hidden platform over 4 consecutive days (acquisition phase). The time that animals spent finding and climbing onto the hidden platform was measured as the escape latency. After finding the platform, each rat was allowed to remain on it for 15 s and was then towel-dried and returned to its home cage. If the rat did not find the platform within 60 s, it was manually guided to the platform and was allowed to remain there for 30 s. A probe trial was performed on day 5, during which the submerged platform was removed and the rats were allowed to freely swim for 60 s. The percentage of time spent in quadrant Q in the probe trial was measured. Each test was recorded using a computer-assisted video tracking system (Techman, Chengdu, China).

### 2.6 Western blotting

The rats were anesthetized and decapitated and brain tissue was collected. The bilateral hippocampus was rapidly dissected, immediately frozen in liquid nitrogen, and stored at −80°C. For western blotting analysis, the hippocampal tissue was homogenized on ice in RIPA buffer plus protease and phosphatase inhibitors (Sigma, St. Louis, MO, USA) and centrifuged at 4°C for 15 min at 12,000 rpm. The protein concentration in the supernatant was determined using a BCA protein assay kit (Beyotime Biotech, Shanghai, China). The protein extracts were denatured by boiling in Laemmli buffer (Bio-Rad, CA, USA). Proteins were separated by SDS–PAGE on 8% resolving gels and were then transferred to PVDF membranes (Millipore, Billerica, MA, USA). The membranes were blocked with 5% nonfat milk at room temperature for 1 h and then incubated at 4°C overnight with primary antibodies targeting DNMT1 (1:1,000; Cell Signaling, Boston, MA, USA), DNMT3b (1:1,000; Abcam, Cambridge, UK), and *β*-actin (1:500; Abcam). After washing, the membranes were incubated with the appropriate HRP-conjugated secondary antibodies. The bands were detected using Pierce ECL Western Blotting Substrate (Thermo Fisher Scientific, Rockford, IL, USA), and their optical density was quantified using ImageJ software (NIH, Bethesda, MD, USA).

### 2.7 Statistical analysis

Data normality was assessed using the Shapiro–Wilk test, and homogeneity of variance was detected using Levene’s test. Statistical analyses were performed using one-way ANOVA with the treatment group (control, PTSD, mTBI, and mTBI + PTSD) as the between-subject factor (Teutsch et al., 2018). Significant main effects were further analyzed using LSD *post hoc* comparisons. For measures taken at repeated time points (MWM training trials), a repeated measures ANOVA was used. Analyses were conducted using SPSS Statistics 29 (IBM Corporation, Armonk, NY, USA) and figures were plotted using GraphPad Prism 10 (GraphPad, La Jolla, CA, USA). Values are presented as means ± standard error of the mean (SEM). *p* < 0.05 were considered statistically significant.

## 3 Results

### 3.1 The effect of mTBI and SPS on the irritability of rats

**Figure 2:**
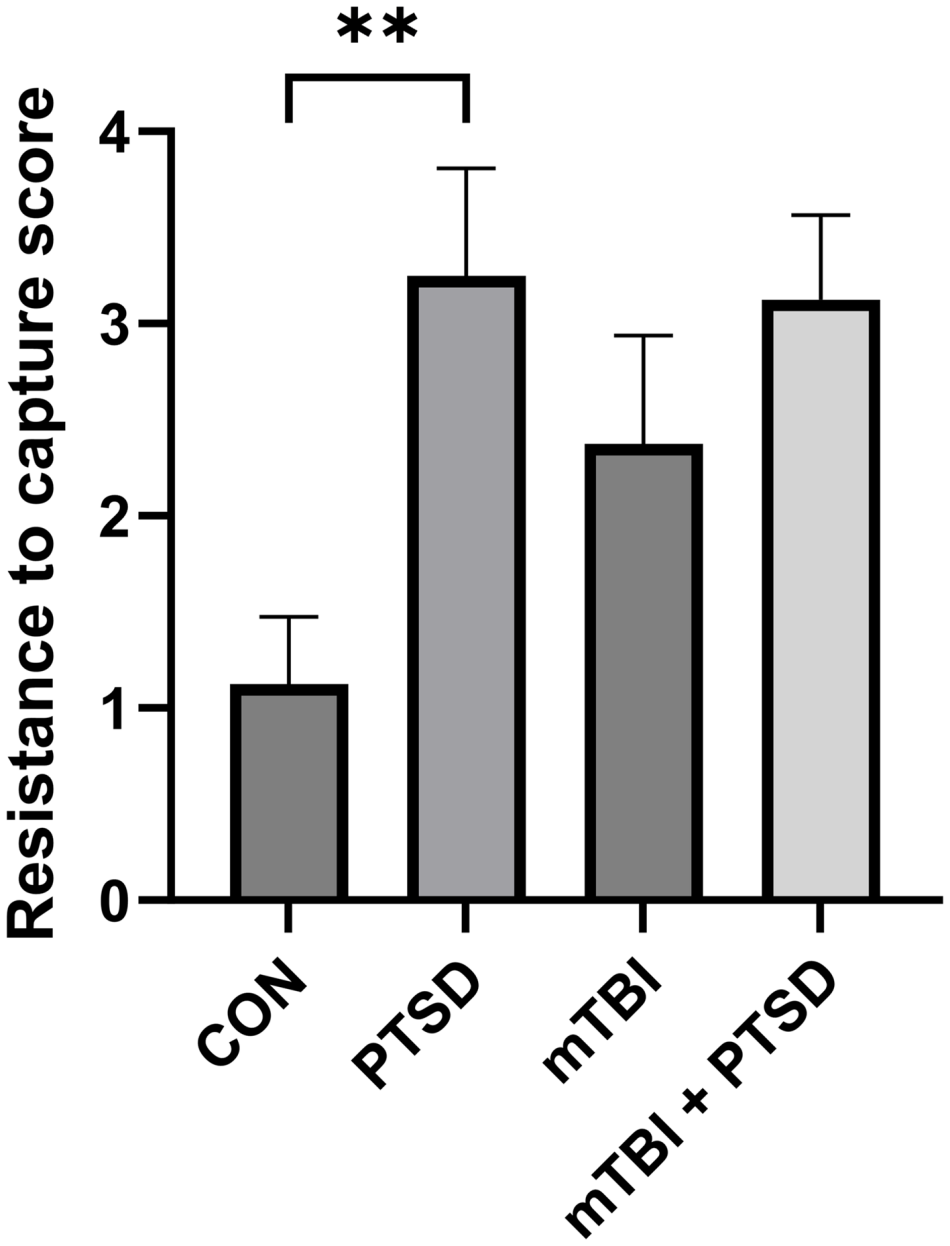
The effect of mTBI and SPS on the irritability of rats in the resistance-to-capture test. Values are presented as means ± SEM of 8 rats per group. CON, control; PTSD, post-traumatic stress disorder; mTBI, mild traumatic brain injury; SPS, single prolonged stress. **p < 0.01.

The RTC test was used to assess the irritability of rats (Figure 2). The higher the score, the greater the irritability. One-way ANOVA revealed a significant main effect across the study groups [*F*_(3, 28)_ = 4.0133; *p* = 0.0171]. Rats in the PTSD group exhibited significantly greater irritability than those in the control group (*p* = 0.0045). There was a trend of greater irritability of rats in the mTBI group compared with the control group, but this effect was not significant (*p* = 0.0803). Meanwhile, rats in the mTBI + PTSD group showed no difference in irritability than those in the PTSD group (*p* = 0.8573).

### 3.2 The effect of mTBI and SPS on anxiety-like behavior and locomotion in rats

**Figure 3:**
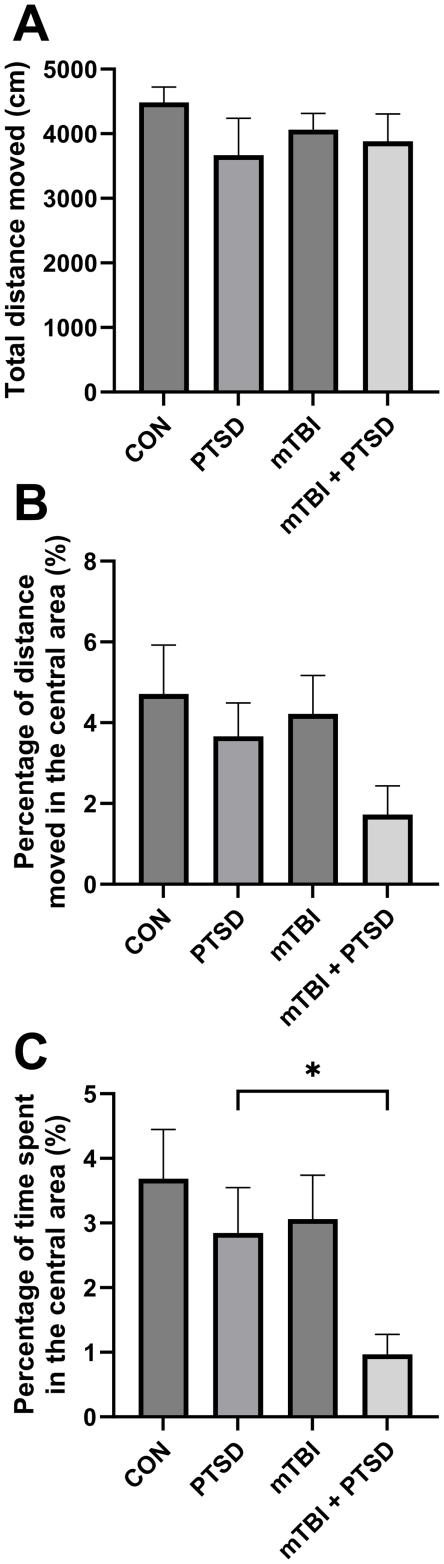
The effect of mTBI and SPS on anxiety-like behavior and locomotion in rats in the open field test. **(A)** Total distance moved. **(B)** Percentage of distance moved in the central area. **(C)** Percentage of time spent in the central area. Values are presented as means ± SEM of 8 rats per group. CON, control; PTSD, post-traumatic stress disorder; mTBI, mild traumatic brain injury; SPS, single prolonged stress. *p < 0.05.

The OFT was performed to assess anxiety levels and locomotor activity in rats (Figure 3). The total distance moved in the open field did not differ among the four groups [*F*_(3, 28)_ = 0.7717; *p* = 0.5196], indicative of similar locomotion potential. For the percentage of time spent in the central area, one-way ANOVA revealed a significant main effect across the study groups [*F*_(3, 28)_ = 3.3548; *p* = 0.0329], with rats in mTBI + PTSD group exhibiting a significantly lower percentage of time spent in the central area compared with those in the PTSD group (*p* = 0.0467), indicating that rats in the mTBI + PTSD group had higher anxiety levels. There was a trend of less time spent in the central area of rats in the PTSD and mTBI groups compared with the control group but with no significant effect (*p* = 0.3625 and *p* = 0.4965, respectively). One-way ANOVA indicated no main effect for the percentage of distance moved in the central area [*F*_(3, 28)_ = 1.9172; *p* = 0.1497], although the four groups showed similar interrelation patterns relative to the percentage of time spent in the central area.

### 3.3 The effect of mTBI and SPS on the depression level of rats

**Figure 4:**
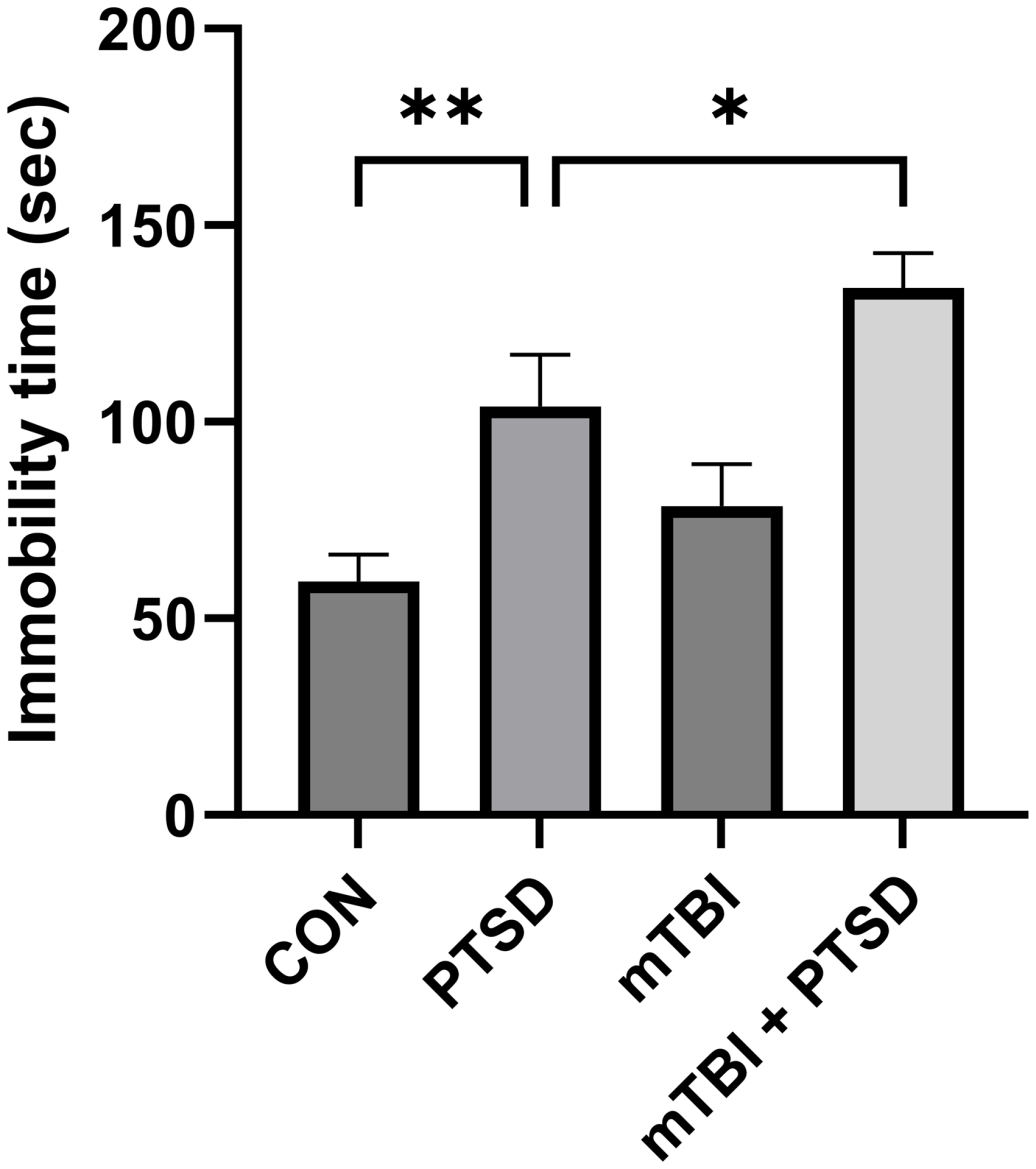
The effect of mTBI and SPS on the depression level of rats in the forced swimming test. Values are presented as means ± SEM of 8 rats per group. CON, control; PTSD, post-traumatic stress disorder; mTBI, mild traumatic brain injury; SPS, single prolonged stress. **p < 0.01, *p < 0.05.

The FST was performed to evaluate the depression level of the rats (Figure 4). The greater the immobility time, the greater the depression level. One-way ANOVA of immobility time revealed a significant main effect across the study groups [*F*_(3, 28)_ = 10.1694; *p* < 0.001]. Rats in the PTSD group exhibited significantly longer immobility time than those in the control group (*p* = 0.0044), indicative of a significantly higher depression level. Compared with the PTSD group, rats in the mTBI + PTSD group showed a significantly higher level of depression (*p* = 0.0445). There was a trend of longer immobility time of rats in the mTBI group compared with the control group; however, this difference was not statistically significant (*p* = 0.1940).

### 3.4 The effect of mTBI and SPS on spatial learning and memory in rats

**Figure 5:**
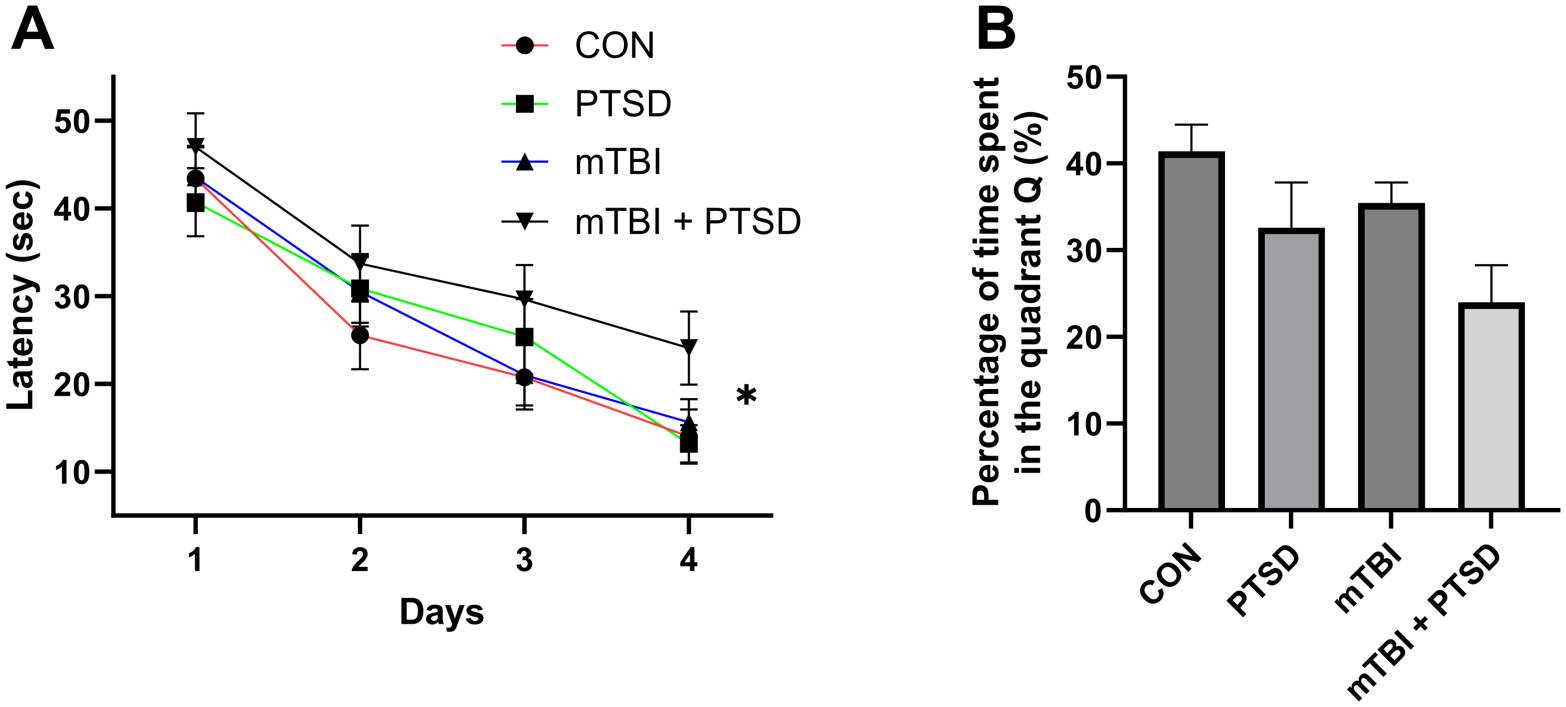
The effect of mTBI and SPS on spatial learning and memory in rats in the Morris water maze test. **(A)** Latency to find the platform during the acquisition of spatial memory (training phase). **(B)** Percentage of time spent in the Q quadrant during the probe trial. Values are presented as means ± SEM of 8 rats per group. CON, control; PTSD, post-traumatic stress disorder; mTBI, mild traumatic brain injury; SPS, single prolonged stress. *p < 0.05.

The effect of mTBI and SPS on spatial learning and memory was evaluated by subjecting rats to the MWM test (Figure 5). Over the course of the 5 testing days, the average swimming speed of rats did not differ among the four groups in each day (all *p* > 0.05). In the spatial memory acquisition training (from day 1 to day 4), a repeated-measures ANOVA for escape latency showed significant main effects of day and treatment [time: *F*_(3, 124)_ = 41.1073; *p* < 0.001; treatment: *F*_(3, 124)_ = 2.7170; *p* = 0.0476], but not for day × treatment interaction [*F*_(9, 124)_ = 0.4041; *p* = 0.9234]. Over the four training days, rats of the mTBI + PTSD group exhibited a significant slower decrease in the latency to find the submerged platform compared with those of CON, PTSD, and mTBI groups (*p* = 0.0091; *p* = 0.0378 and *p* = 0.0422, respectively), indicating that spatial learning ability was impaired in mTBI + PTSD-treated rats. In the probe trial test on day 5, one-way ANOVA for the percentage of time spent in quadrant Q revealed a significant main effect across the study groups [*F*_(3, 28)_ = 3.4501; *p* = 0.0298]. Compared with the PTSD group, rats in the mTBI + PTSD group showed a trend toward less time in the Q quadrant, suggestive of impaired spatial memory; nevertheless, the difference was not statistically significant (*p* = 0.1296). Rats in the PTSD and mTBI groups showed a trend toward less time in quadrant Q than rats in the control group, but this difference was also not significant (*p* = 0.1216 and *p* = 0.2873, respectively).

### 3.5 The effects of mTBI and SPS on hippocampal DNMT1 and DNMT3b protein expression levels in rats

**Figure 6:**
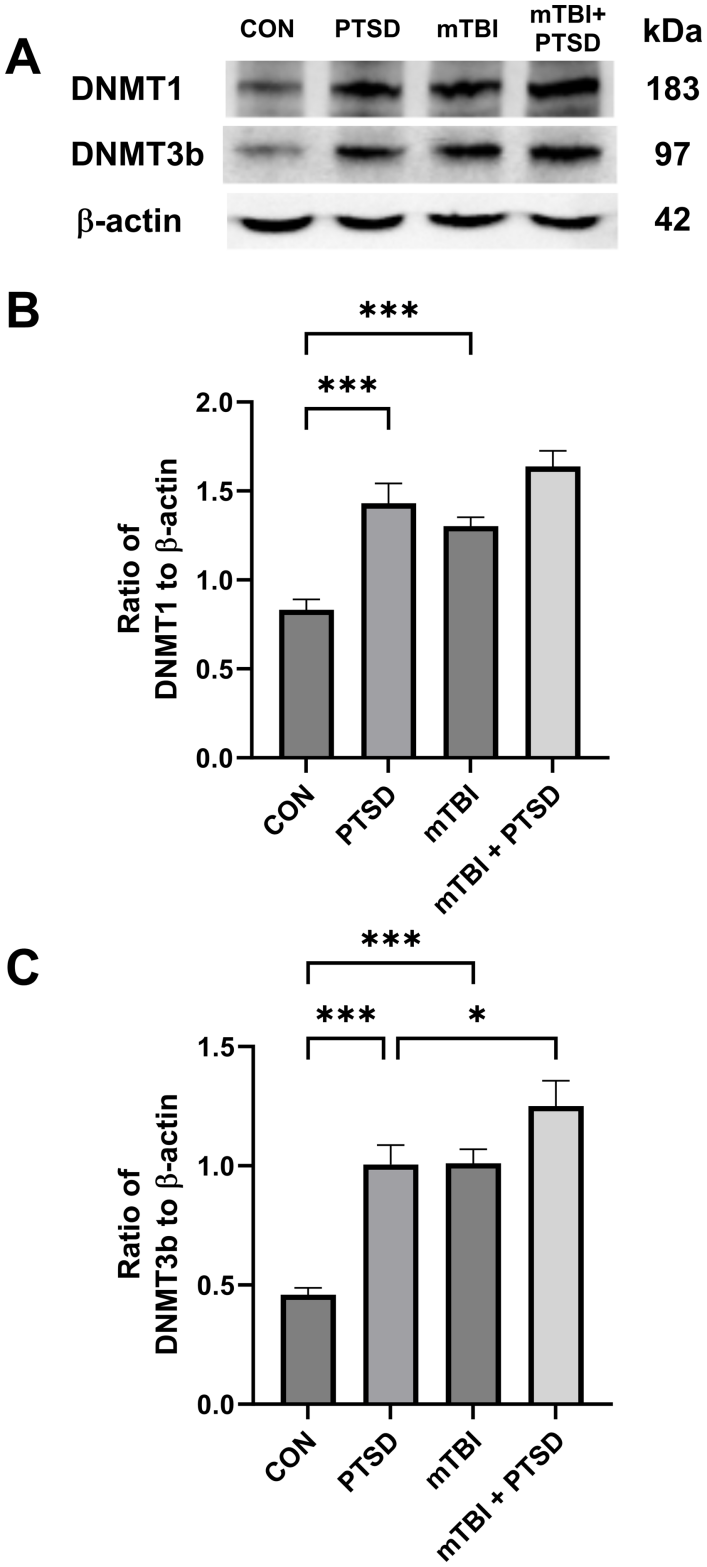
The effect of mTBI and SPS on hippocampal DNMT1 and DNMT3b protein levels in rats. **(A)** Representative western blots of DNMT1, DNMT3b, and β-actin. **(B)** The level of DNMT1 relative to β-actin. **(C)** The level of DNMT3b relative to β-actin. The order of the western blot panels in **(B)** and **(C)** is the same as that in **(A)**. Values are presented as means ± SEM of 8 rats per group. CON, control; PTSD, post-traumatic stress disorder; mTBI, mild traumatic brain injury; SPS, single prolonged stress. ***p < 0.001, *p < 0.05.

Western blotting was used to assess the protein expression of DNMT1 and DNMT3b in the hippocampus of rats (Figure 6). One-way ANOVA revealed a significant main effect across the study groups for DNMT1 [*F*_(3, 20)_ = 17.8762; *p* < 0.001] and DNMT3b [*F*_(3, 20)_ = 20.1635; *p* < 0.001]. Rats in the PTSD group exhibited significantly higher hippocampal DNMT1 and DNMT3b protein expression levels (both *p* < 0.001) than those in the control group. Rats in the mTBI group also displayed significantly higher hippocampal protein levels of DNMT1 and DNMT3b (both *p* < 0.001) than rats in the control group. Compared with animals in the PTSD group, those in the mTBI + PTSD group exhibited significantly higher hippocampal DNMT3b protein levels (*p* = 0.0313). There was a trend of increased expression of DNMT1 in the hippocampus of rats in the mTBI + PTSD group compared with the PTSD group, although not significantly (*p* = 0.0844).

## 4 Discussion

In the current study, we demonstrated that mTBI increases vulnerability to PTSD in rats. Compared with the PTSD group, rats in the mTBI + PTSD group exhibited higher anxiety levels in the OFT, higher depression levels in the FST, and impaired spatial learning and memory in the MWM test. Rats in the mTBI + PTSD group also exhibited increased hippocampal protein expression of DNMT3b compared with the PTSD group, indicating that hippocampal DNA methylation may play a role, at least in part, in the promotive effect of mTBI on vulnerability to PTSD.

PTSD is a major mental disorder caused by exposure to traumatic stress (Stein et al., 2021). The comorbidity between mTBI and PTSD has become a major focus of clinical research (Van Praag et al., 2019; Zuckerman et al., 2019; Loignon et al., 2020). Most TBIs (70–90%) are classified as mild (Maas et al., 2017). Many patients with mTBI do not fully recover from their injury, with up to 20% developing PTSD (Stein et al., 2019; Howlett et al., 2022). The overlapping symptoms of mTBI and PTSD, along with their clinical heterogeneity, complicate diagnostic differentiation and effective intervention (Huang et al., 2016; Algamal et al., 2019). Studies have shown that patients with mTBI are at an increased risk for PTSD, suggesting that mTBI increases the vulnerability for subsequent PTSD development (Loignon et al., 2020; van der Vlegel et al., 2021). Moreover, patients with PTSD and a history of mTBI often exhibit more severe PTSD symptoms and more pronounced neurological changes than those who have not sustained a mTBI (Vanderploeg et al., 2009; Lindemer et al., 2013; Spielberg et al., 2015). mTBI may decrease cognitive reserve, making individuals more susceptible to the effects of PTSD-related neuropathology (Stein and McAllister, 2009). For instance, one study proposed that mTBI may exacerbate the impact of brain microstructure on PTSD symptoms, especially within stress-vulnerable brain regions (i.e., the limbic/paralimbic system) (Sydnor et al., 2020). The mechanisms underlying the association between mTBI and subsequent PTSD onset may include mechanical damage to white matter tracts, neuroinflammation, and stress-related oxidative damage (Stein and McAllister, 2009; Gordon et al., 2018; Kaplan et al., 2018), which warrant further exploration.

Preclinical animal studies can contribute considerably to the determination of the mechanisms underpinning the interaction between mTBI and PTSD (Algamal et al., 2019). However, preclinical animal studies exploring this area of research are scarce (Ojo et al., 2014). One study demonstrated that repetitive concussive TBI, combined with post-injury foot shock stress in mice, worsened social and depression-like behaviors (Klemenhagen et al., 2013). A different study reported heightened behavioral impairment and hippocampal neuroinflammation in a mouse model of co-morbid TBI and PTSD (Fesharaki-Zadeh et al., 2020). Additionally, mice subjected to both TBI and SPS showed significant gait and conditioned fear impairments (Teutsch et al., 2018). In adult rats, meanwhile, exposure to mTBI, concurrent with social defeat stress, led to exaggerated anxiety and contextual fear extinction impairment (Davies et al., 2016). Consistent with these findings, our current work also showed that rats subjected to both mTBI and PTSD exhibited higher anxiety levels in the OFT, greater levels of depression in the FST, and more pronounced spatial learning and memory impairment in the MWM test than those with simulated PTSD. Taken together, these findings from animal studies support that mTBI increases vulnerability to subsequent PTSD development.

Several models, including fluid percussion, controlled cortical impact, blast, and weight drop, have been developed to investigate the consequences of mTBI in rodents (Namjoshi et al., 2013). The weight-drop injury model induces non-penetrating, diffuse injury, simulating the rotational acceleration or deceleration experienced by the brain during traumatic events (Mychasiuk et al., 2014). In our study, we employed a mild, closed-head, weight-drop injury to model mTBI in rats. We found that there was a trend of higher anxiety levels in the OFT, greater levels of depression in the FST, and impaired spatial learning and memory in the MWM test in the mTBI group compared with the control group. However, these effects did not reach statistical significance, suggesting that the intensity of the TBI induced in our study was indeed mild and was likely at the very low end of the mTBI continuum. The exact duration of increased cerebral vulnerability after injury is currently unknown (Kawa et al., 2018). In this study, we chose to administer SPS 72 h post-mTBI, and our results indicated that mTBI increases vulnerability to PTSD in rats during this time window.

However, some studies have reported controversial findings. For instance, one study showed that combined exposure to repetitive mTBI and chronic stress resulted in an apparent amelioration of stress-related behaviors in the cued fear memory and forced swimming tests at the 3-month time point (Algamal et al., 2019). In another rodent model of mTBI with stress, mTBI abolished contextual, but not cue fear conditioning, as elicited in a PTSD model (Ojo et al., 2014). These discrepancies may be attributable to differences in the mTBI and PTSD models employed, the order in which mTBI and PTSD were induced, differences in the strains and ages of rodents, and variations in the time points when behavioral tests were performed. Further studies are warranted to clarify the elusive relationship between mTBI and PTSD.

Research suggests that the hippocampus might play an important role in the course of mTBI and PTSD (Kaplan et al., 2018; Rowland et al., 2021). For instance, weight drop-induced mTBI has been shown to enhance cell death and reduce neuron numbers in the hippocampus, effects that may partially explain the heightened anxiety and contextual fear conditioning observed following mTBI (Meyer et al., 2012). In humans, hippocampal volume has been associated with the risk for PTSD development (Stein et al., 2021), with patients who have PTSD exhibiting smaller hippocampal volumes than those without the condition (Chen et al., 2018; Logue et al., 2018). One study suggested that reduced hippocampal volume is a vulnerability factor for developing PTSD (Bolsinger et al., 2018). Furthermore, structural and functional changes in the hippocampus have been observed in rodent models of PTSD (Schneider et al., 2016).

DNA methylation is a highly stable, yet reversible, epigenetic modification, governed by a dynamic equilibrium between the activities of DNA methyltransferases (DNMTs) and demethylases (Stricker and Götz, 2018). DNA methylation in the hippocampus is implicated in the development of mTBI and PTSD (Nielsen et al., 2019; Balasubramanian et al., 2021). Accordingly, in the current study, we investigated the possible role of DNA methylation in the hippocampus in the increased vulnerability to PTSD induced by mTBI. The DNMT3b mRNA level was reported to be upregulated in the hippocampus of rats 14 days after exposure to blast injury (Bailey et al., 2015). In mice, fear conditioning increased DNMT3a expression in the hippocampus, while DNMT inhibition suppressed hippocampal-dependent fear learning (Elliott et al., 2016). One study reported that the expression levels of DNMT3a and DNMT3b were upregulated in the hippocampus of adult rats following contextual fear conditioning (Miller and Sweatt, 2007). In another study, it was found that mice subjected to foot shock displayed significant increases in DNMT3a and DNMT3b levels in the hippocampus (Ju et al., 2017). In line with these findings, we observed that rats in both the mTBI and PTSD groups had higher hippocampal protein levels of DNMT1 and DNMT3b than rats in the control group. Additionally, rats in the mTBI + PTSD group presented higher hippocampal DNMT3b protein levels than those in the PTSD group. These results indicated that hippocampal DNA methylation might play a role in the mechanism through which mTBI increases vulnerability to PTSD. Further research is needed to explore the underlying mechanisms in greater depth.

This study had some limitations. First, we only included male rats. Rodent models of PTSD showed an increased vulnerability in females (Whitaker et al., 2014). Comparisons between male and female rats should be incorporated in future studies of comorbid mTBI and PTSD. Second, the time interval between mTBI and PTSD induction in this study was set at 3 days. The effects reported in this study may vary with different time intervals or changes in the order of mTBI and PTSD induction. Finally, we only investigated the protein levels of DNMT1 and DNMT3b in the hippocampus using western blotting. Future studies should check the expression and localization of DNMT1 and DNMT3b and assess DNA methylation patterns for 5hmC and 5mC in the hippocampus of rats by immunohistochemistry. Also, future studies should examine global DNA methylation levels in the hippocampus or the methylation of promotor regions of genes that may be involved in the interaction between mTBI and PTSD.

## Data Availability

The raw data supporting the conclusions of this article will be made available by the authors, without undue reservation.
