# Supplementary material for: Mild traumatic brain injury increases vulnerability to post-traumatic stress disorder in rats and the possible role of hippocampal DNA methylation
Source: Front Behav Neurosci. 2025 Mar 3;19:1539028. doi: 10.3389/fnbeh.2025.1539028 (PMC11911326; doi:10.3389/fnbeh.2025.1539028)
Supplement: Supplementary file 1 [file Data_Sheet_1.docx]

**Graphic abstract.** Created with BioRender.com. Images reproduced with permission from Verbitsky et al., 2020.

Verbitsky, A., Dopfel, D., and Zhang, N. (2020). Rodent models of post-traumatic stress disorder: behavioral assessment. *Transl. Psychiatry* 10, 132. doi: 10.1038/s41398-020-0806-x
