# Supplementary figures and images for: Mild traumatic brain injury increases vulnerability to post-traumatic stress disorder in rats and the possible role of hippocampal DNA methylation
Source: Front Behav Neurosci. 2025 Mar 3;19:1539028. doi: 10.3389/fnbeh.2025.1539028 (PMC11911326; doi:10.3389/fnbeh.2025.1539028)

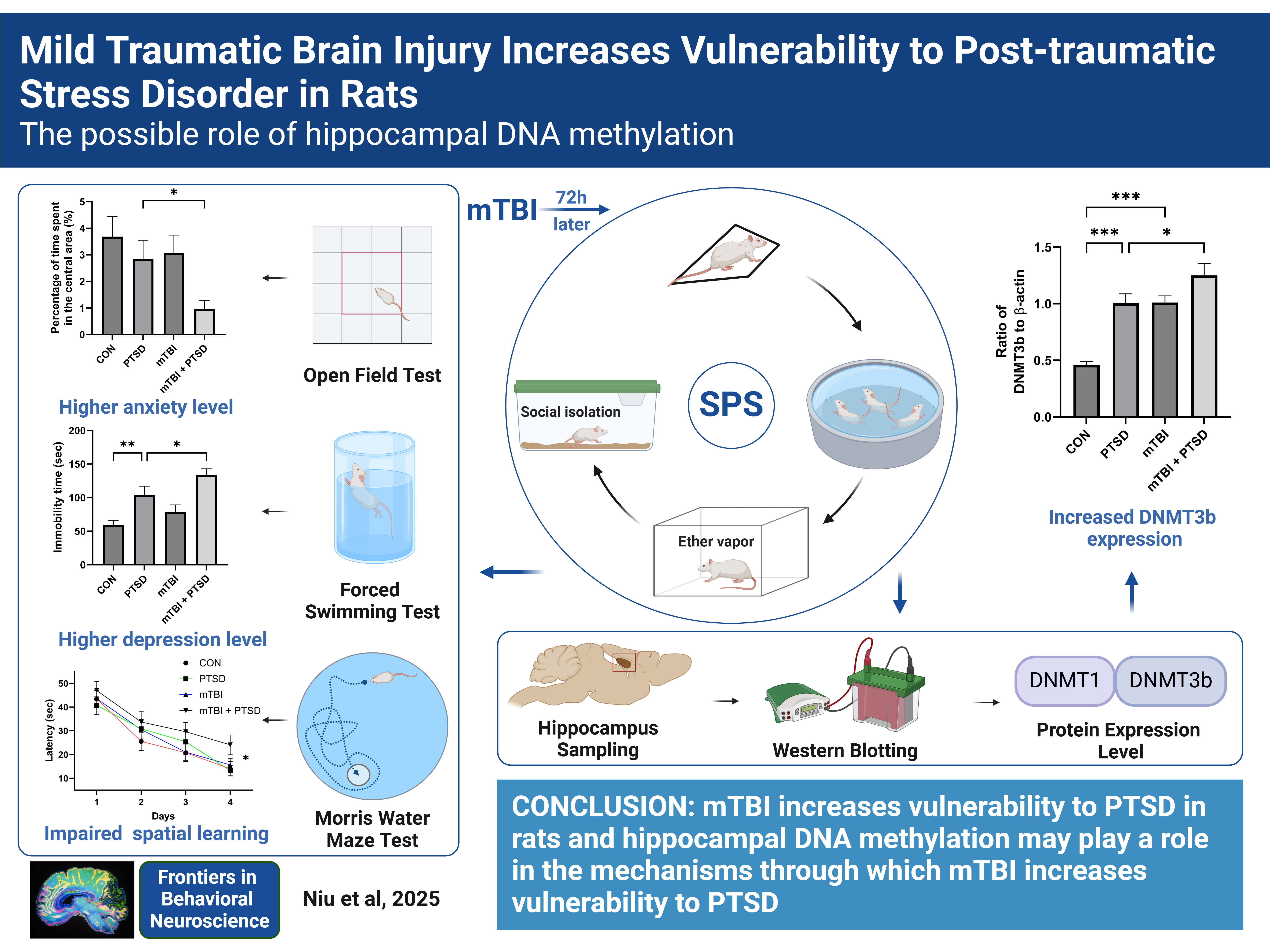

Supplement: Supplementary file 2 [file Image_1.jpeg]
